# Resolving data bias improves generalization in binding affinity prediction

**DOI:** 10.1038/s42256-025-01124-5

**Published:** 2025-10-21

**Authors:** David Graber, Peter Stockinger, Fabian Meyer, Siddhartha Mishra, Claus Horn, Rebecca Buller

**Affiliations:** 1 Seminar for Applied Mathematics, Department of Mathematics and ETH AI Center, Zurich, Switzerland; 2 Competence Center for Biocatalysis, Zurich University of Applied Sciences, Waedenswil, Switzerland; 3 Institute for Computational Life Sciences, Zurich University of Applied Sciences, Waedenswil, Switzerland; 4 Department of Chemistry, Biochemistry and Pharmaceutical Sciences, University of Bern, Bern, Switzerland; 5 Yale School of Medicine, Department for Bioinformatics and Data Science, New Haven, CT USA

**Keywords:** Drug discovery, Scientific data, Machine learning, Cheminformatics

## Abstract

The field of computational drug design requires accurate scoring functions to predict binding affinities for protein–ligand interactions. However, train–test data leakage between the PDBbind database and the Comparative Assessment of Scoring Function benchmark datasets has severely inflated the performance metrics of currently available deep-learning-based binding affinity prediction models, leading to overestimation of their generalization capabilities. Here we address this issue by proposing PDBbind CleanSplit, a training dataset curated by a new structure-based filtering algorithm that eliminates train–test data leakage as well as redundancies within the training set. Retraining current top-performing models on CleanSplit caused their benchmark performance to drop substantially, indicating that the performance of existing models is largely driven by data leakage. By contrast, our graph neural network model maintains high benchmark performance when trained on CleanSplit. Leveraging a sparse graph modelling of protein–ligand interactions and transfer learning from language models, our model is able to generalize to strictly independent test datasets.

## Abstract (web summary)

Graber et al. characterize biases and data leakage in protein–ligand datasets and show that a cleanly filtered training–test split leads to improved generalization in binding affinity prediction tasks.

## Main

Structure-based drug design (SBDD) aims to design small-molecule drugs that bind with high affinity to specific protein targets. In recent years, deep neural networks have begun to revolutionize the field, offering new possibilities for computational drug design. These include new protein folding models such as RoseTTAFold All-Atom^1^, AlphaFold3^2^ and Boltz-1^3^, which can also consider small-molecule ligands to predict potential binding conformations. Furthermore, generative artificial intelligence can design entirely new protein–ligand interactions. For example, RFdiffusion^4^ can construct proteins around small molecules starting from random clouds of amino acids whereas the denoising diffusion model DiffSBDD^5^ generates new ligands tailored to fit specific protein pockets. Although these methods excel at generating diverse collections of protein–ligand interactions, these interactions are not necessarily characterized by drug-like affinity. Therefore, using these models for development of small-molecule drugs requires scoring functions that can accurately predict the absolute binding affinities for protein–ligand poses and identify high-affinity complexes. Classical scoring functions, such as force-field-based, empirical and knowledge-based methods implemented in docking tools such as AutoDock Vina^6^ and GOLD^7^ are computationally intensive and show limited accuracy in binding affinity prediction^8–11^. Despite notable advancements in deep-learning-based scoring functions, including the design of many convolutional^12–23^ and graph neural networks^24–32^, accurately predicting binding affinities for protein–ligand poses remains an outstanding challenge.

In addition to the fact that many deep-learning-based scoring functions are either not publicly available or are difficult to implement, the key reason for their limited applicability currently is the observation that these scoring functions perform poorly, with considerably lower than expected accuracy on independent test datasets^33–36^. This large gap between benchmark and real-world performance has been attributed to the underlying training and evaluation procedures used for the design of these scoring functions. Typically, these models are trained on the PDBbind database^37,38^, and their generalization is assessed using the comparative assessment of scoring function (CASF) benchmark datasets^10^. However, several studies have reported a high degree of similarity between PDBbind and the CASF benchmarks. Owing to this similarity, the performance on CASF overestimates the generalization capability of models trained on PDBbind^10,39,40^. Alarmingly, some of these models even perform comparably well on the CASF datasets after omitting all protein or ligand information from their input data. This suggests that the reported impressive performance of these models on the CASF benchmarks is not based on an understanding of protein–ligand interactions. Instead, memorization and exploitation of structural similarities between training and test complexes appear to be the main factors driving the observed benchmark performance of these models^35,36,41–43^.

Our first goal in this paper is to further investigate the presence of a train–test data leakage between PDBbind and the commonly used CASF benchmarks. To this end, we propose a new structure-based clustering algorithm to analyse and filter datasets of protein–ligand complex structures. By identifying large similarities between PBDbind and CASF datasets, our algorithm revealed a substantial level of train–test data leakage. Going further, it also enabled us to devise a new split for the PBDbind dataset, providing a better set-up for the training and testing of structure-based affinity prediction models. Our filtered training dataset, termed PDBbind CleanSplit, is strictly separated from the CASF benchmark datasets, turning them into true external datasets and enabling genuine evaluation of model generalizability.

To evaluate the true performance of recently published deep-learning-based scoring functions, we retrained the state-of-the-art binding affinity prediction models GenScore^30^ and Pafnucy^12^ on the PDBbind CleanSplit dataset with reduced data leakage. Although these models had previously shown excellent benchmark performance when trained on the original PDBbind dataset, their performance dropped markedly when trained on PDBbind CleanSplit, confirming that the previous high scores were largely driven by data leakage.

Recognizing that the generalization capability of existing deep-learning-based scoring functions might be much lower than previously thought, our second goal in this paper was to design a binding affinity prediction model with robustly validated generalization capability. To this end, we combined a new graph neural network (GNN) architecture with transfer learning from large language models and trained this model on the filtered PDBbind CleanSplit. Despite the reduced data leakage, our graph neural network for efficient molecular scoring (GEMS) achieves state-of-the-art predictions on the CASF benchmark. Because all protein–ligand complexes that remotely resembled any from the CASF test set were excluded from training, we can confidently state that the performance of GEMS is not the result of exploiting data leakage, but genuinely reflects its capability to generalize to new complexes. Moreover, our ablation studies showed that GEMS fails to produce accurate predictions when protein nodes are omitted from the graph, suggesting that its predictions are based on a genuine understanding of protein–ligand interactions.

GEMS is a promising tool with broad potential impact in the field of SBDD. Generative models such as RFdiffusion and DiffSBDD can generate libraries of new protein–ligand interactions, but their potential in drug design has been bottlenecked by the lack of accurate models to predict binding affinities for these interactions. GEMS fills this critical gap in SBDD. With its robust generalization capabilities evaluated on strictly independent datasets, it provides the prediction accuracy needed to identify interactions with therapeutic potential. To enable researchers to leverage and further develop GEMS, we have made all Python code publicly available in an easy-to-use format.

## Results

### PDBbind dataset filtering

**Fig. 1:**
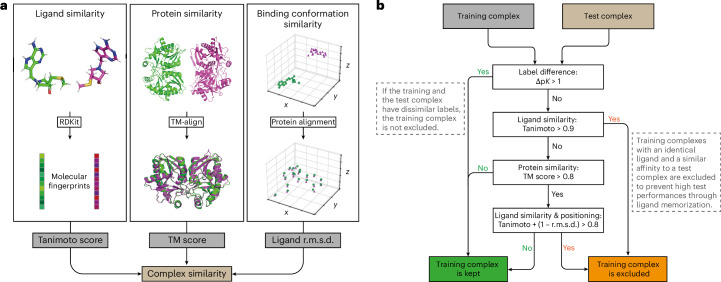
Overview of the similarity computation between two protein–ligand complexes. **a**, Our structure-based dataset filtering algorithm evaluates structural similarity of protein–ligand complexes using a combination of TM scores, Tanimoto scores and pocket-aligned ligand r.m.s.d. The Tanimoto scores identify chemically similar ligands and range from 0 (no similarity) to 1 (identical). TM scores are computed with TM-align, a tool that compares protein structures by finding the optimal alignment of their three-dimensional structures and outputs a score ranging from 0 (no similarity) to 1 (identical). This score identifies proteins with high structural similarity, even when sequence identity is low (for example, when one protein is a substructure of the other). Pocket-aligned ligand r.m.s.d. scores compare the positioning of ligands within aligned protein pockets. Ligands are transformed into the same coordinate frame using the optimal alignment from TM-align, and an r.m.s.d. calculation provides a quantitative measure of positional similarity. **b**, Decision tree showing the exclusion criteria and the information flow of the filtering algorithm when comparing a training and a test complex. The first layer of the algorithm compares the affinity labels of the complexes (p*K* values; see Methods). Training complexes with high structural similarity but different activity are not excluded, to avoid excluding data points that can provide valuable insights into activity cliffs. The second layer excludes all training complexes with similar affinity and identical ligand (Tanimoto > 0.9) to make the test complex ligands unique and avoid successful predictions through ligand memorization. The third and fourth layer exclude training complexes based on protein similarity and a combined assessment of ligand and binding conformation similarity. This four-layer approach identifies complexes with similar interaction patterns, even when traditional sequence-based methods would overlook these similarities.

To gain the ability to identify and remove structural similarities in datasets of protein–ligand complexes, we set out to design a structure-based clustering algorithm (Fig. 1). In this algorithm, the computation of similarity between two protein–ligand complexes is based on a combined assessment of protein similarity (TM scores^44^), ligand similarity (Tanimoto scores^45^) and binding conformation similarity (pocket-aligned ligand root-mean-square deviation (r.m.s.d.)). Combining these three metrics allows a robust and detailed comparison of protein–ligand complex structures. Importantly, unlike traditional sequence-based analysis approaches, our multimodal filtering can identify complexes with similar interaction patterns, even when the proteins have low sequence identity (Supplementary Fig. 1).

**Fig. 2:**
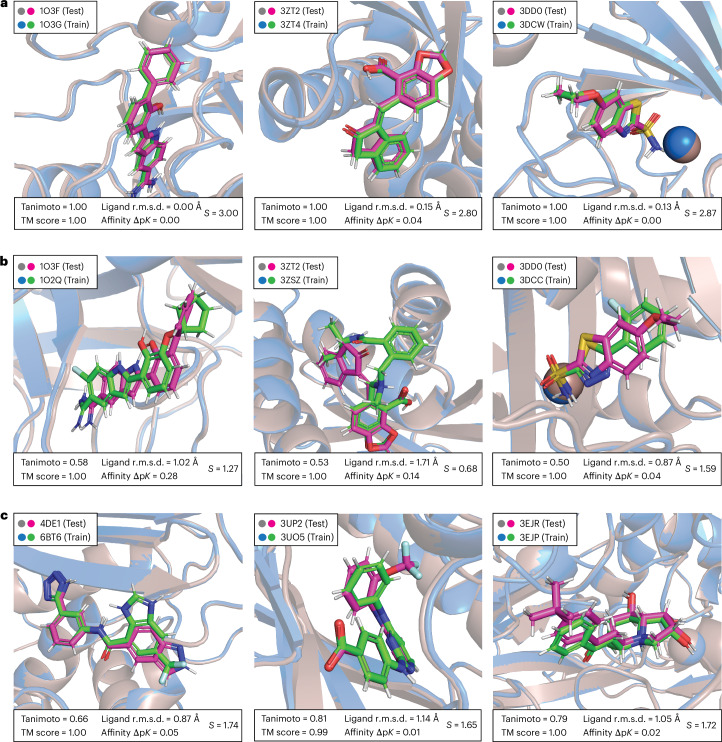
Superpositions of complexes highlighting train–test structural similarities before and after filtering. **a**, Superpositions of the most prominent train–test similarities before applying the filtering algorithm. **b**, Superpositions of the same test complexes as in **a**, now shown with the most similar training complexes found in PDB CleanSplit. **c**, Superpositions of the closest train–test similarities that remained postfiltering in the dataset PDBbind CleanSplit. Protein structures from the test and training datasets are depicted as grey and blue cartoons, respectively, with ligands shown in magenta (test) and green (train). Below each superposition, the Tanimoto score, TM score, ligand r.m.s.d. and affinity difference (Δp*K*) is shown, which are combined into an overall similarity score *S* = TM score + Tanimoto + (1 − ligand r.m.s.d.) − Δp*K*. This *S* score was computed for all possible train–test pairs and served to select representative complexes for this figure. For each training dataset, the depicted pairs were selected from the top five pairs with the highest *S*, prioritizing good ligand visibility. In some superpositions (1O3F/1O3G and 3DD0/3DWC), one structure has been slightly shifted to improve visibility. Original PyMol sessions of all of superpositions are provided on GitHub.

By comparing all CASF complexes with all PDBbind complexes, we identified a large number of train–test pairs with exceptionally high similarity, sharing not only similar ligand and protein structures but also comparable ligand positioning within the protein pocket and, unsurprisingly, also being accompanied by closely matched affinity labels (Fig. 2a). Consequently, these structures provide nearly identical input data points to the model, enabling accurate prediction of test data point labels through simple memorization. Using the thresholds of our filtering algorithm, nearly 600 such similarities were detected between PDBbind training and CASF complexes, involving 49% of all CASF complexes. These findings reveal a clear train–test data leakage when models are trained on PDBbind and tested on the CASF benchmark datasets, with nearly half of the CASF complexes not presenting new challenges to these models.

Our filtering algorithm reduced train–test data leakage by excluding all training complexes that closely resemble any CASF test complex (Fig. 1b). In addition, it removed all training complexes with ligands identical to those in the CASF test complex (Tanimoto > 0.9), ensuring that the ligands in the test datasets are never encountered with similar affinity during model training. This step provides an additional safeguard against ligand-based data leakage, addressing previous research that shows that GNNs for binding affinity predictions often rely on ligand memorization to make affinity predictions^41^. Together, this filtering excluded 4% of all training complexes. The remaining train–test pairs with the highest similarity after filtering exhibited clear structural differences (Fig. 2c), highlighting the effectiveness of our filtering algorithm in removing structurally similar data points. The resulting filtered training dataset is strictly separated from the CASF datasets, allowing models trained on it to be evaluated on the CASF benchmark and offering a genuine assessment of their generalization to unseen protein–ligand complexes.

In addition to the train–test overlap, we found numerous similarity clusters within the training dataset itself. According to the thresholds of our filtering algorithm, nearly 50% of all training complexes are part of a similarity cluster. This means that random splitting inadvertently leads to inflated validation performance metrics, as some validation complexes can be predicted by matching labels with similar training complexes. Consequently, it is not surprising that models trained on a dataset with such extensive redundancies perform structure-matching, thereby settling for an easily attainable local minimum in the loss landscape. We hypothesized that this redundancy hampers model generalization as it encourages memorization, leading to models that rely on exploiting structural similarities. Thus, we proposed that binding affinity prediction models would benefit from a more diverse dataset as a robust basis for training. To test this hypothesis, our filtering algorithm includes a step to reduce training dataset redundancy. To find an optimal trade-off between maximizing dataset size and minimizing redundancy, we used adapted filtering thresholds to identify and eliminate the most striking similarity clusters (Methods). Using these adapted thresholds, our filtering algorithm iteratively removed complexes from the training dataset until all similarity clusters were resolved. Ultimately, this process resulted in the removal of another 7.8% of all training complexes.

Given the extensive data leakage between PDBbind and the CASF benchmarks, the performance reported by many published models trained on these datasets is likely to overestimate their true generalization capabilities. Combining our strategies for reducing train–test data leakage and minimizing training dataset redundancy (Methods: ‘Filtering algorithm’), we have created a new refined split of the PDBbind dataset, which we call PDBbind CleanSplit.

### Search algorithms

To illustrate the effect of train–test data leakage on model performance, we devised a simple algorithm that predicts the affinity of each CASF test complex by identifying the five most similar training complexes and averaging their affinity labels. This algorithm showed competitive CASF2016 prediction root-mean-square error (r.m.s.e.) compared with some published deep-learning-based scoring functions (Pearson *R* = 0.716, r.m.s.e. = 1.517). To explore whether ligand memorization alone is sufficient for accurate CASF predictions, we modified the algorithm to search for the five training complexes with the most similar ligands. Averaging the labels of these complexes produced similarly high performance (Pearson *R* = 0.707, r.m.s.e. = 1.539), confirming previous research on the importance of ligand memorization^41^ (Table 1).

**Table 1:**
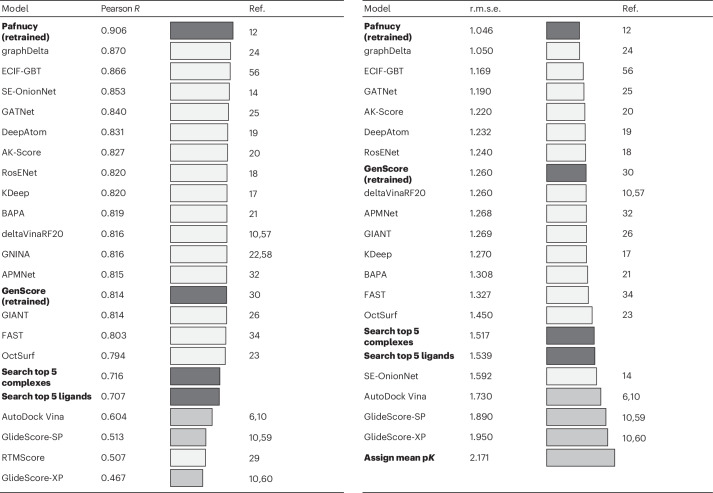
Comparison of model performance^56–60^

Reported CASF2016 scoring performance (Pearson correlation coefficients and r.m.s.e. values) of all published deep-learning-based scoring functions that have, to our knowledge, evaluated their scoring performance on the complete CASF2016 (*n* = 285) dataset. Models with dark grey bars and bold text have been trained or created in this study (Pafnucy, GenScore and our two search algorithms ‘Search top 5 complexes’ and ‘Search top 5 ligands’). White bars represent published binding affinity prediction models, with performance values taken from literature. Models with light-grey bars are classical scoring functions (AutoDock Vina, GlideScore). As a baseline, the bottom bar in the r.m.s.e. table shows the error that is achieved when the average training dataset label is assigned to all CASF2016 complexes.

Source data

**Fig. 3:**
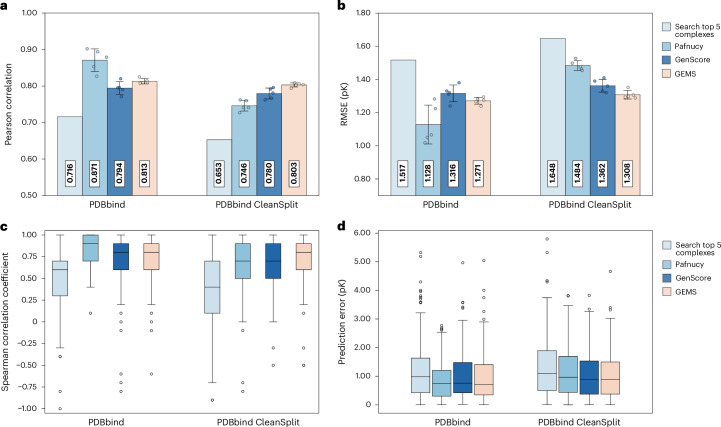
Prediction accuracy of GEMS, Pafnucy and GenScore in dependence of dataset filtering. **a**, CASF2016 Pearson correlation coefficients of a simple search algorithm (Search top 5 complexes), Pafnucy, GenScore and GEMS when trained on the original PDBbind dataset (left) and the filtered PDBbind CleanSplit dataset (right). **b**, CASF2016 prediction r.m.s.e. values of a simple search algorithm (Search top 5 complexes), Pafnucy, GenScore and GEMS when trained on the original PDBbind dataset (left) and the filtered PDBbind CleanSplit dataset (right). **c**, Ranking power: the distribution of Spearman correlation coefficients for the search algorithm, Pafnucy, GenScore and GEMS across the 57 independent clusters in CASF2016 (*n* = 57, unit = cluster), presented separately for training on PDBbind (left) and PDBbind CleanSplit (right). **d**, Distribution of prediction errors (pK unit) across the 285 CASF2016 complexes (*n* = 285, unit = protein–ligand complex) shown for a simple search algorithm (Search top 5 complexes), Pafnucy, GenScore and GEMS when trained on the original PDBbind dataset (left) and the filtered PDBbind CleanSplit dataset (right). For each Pafnucy, GenScore and GEMS, five models were trained with five different train–validation splits. The performance values in **a** and **b** reflect the mean CASF2016 performance of the five models (*n* = 5 independently trained models), with dots showing the individual values and error bars representing the standard deviation across the performance of the five models. To compute the distributions in **c** and **d**, the predictions of the five models were averaged into an ensemble prediction for each of the 285 CASF2016 complexes. Boxplots show the median (centre line), 25th–75th percentiles (box) and whiskers extending to data points within 1.5 × IQR; outliers are shown individually. Source data

When using the two search algorithms on the filtered PDBbind CleanSplit, we found that this led to a dramatic drop in their CASF prediction performance. Averaging the affinity label of the five most similar training complexes resulted in an r.m.s.e. of 1.648 and a Pearson correlation of 0.653. Using the labels of the five complexes with the most similar ligands resulted in an r.m.s.e. of 1.711 and a Pearson correlation of 0.625 (Fig. 3a,b).

Overall, the success of these two search algorithms on the unfiltered PDBbind illustrates how much training data memorization can boost CASF prediction accuracy when training on PDBbind. The low accuracy of the same algorithms on PDBbind CleanSplit, however, demonstrates that the train–test similarities have been largely removed through our filtering. For models trained on PDBbind CleanSplit, training data memorization is not sufficient for high CASF performance.

### Retraining state-of-the-art models

Building on the findings we obtained with our simple search algorithms, we set out to investigate whether the train–test data leakage within PDBbind has similarly inflated the benchmark performance of published state-of-the-art models. Toward this goal, we began retraining the top-performing models to reproduce their reported results and evaluate their performance when trained on PDBbind CleanSplit. Unfortunately, we encountered major obstacles that made reproducing most of these models virtually impossible. These obstacles included the absence of public code repositories, the availability of code that supported inference only, a lack of training instructions and reliance on refined, augmented, or proprietary datasets that are not publicly accessible. However, retraining was successful for two models, GenScore^30^ and the well-known Pafnucy binding affinity prediction model^12^. Pafnucy, published in 2018, was originally trained on the 2016 version of PDBbind, and its benchmark performance has been surpassed by newer models over time. We retrained Pafnucy using a five-fold cross-validation approach on the more recent 2020 version of PDBbind according to the authors’ instructions. This increased Pafnucy’s CASF performance to an r.m.s.e. of 1.046, making it the best-performing binding affinity prediction models evaluated on the complete CASF2016 dataset (Table 1). As a second test case, we retrained GenScore^30^, a recent graph-based model that reports similarly high performance in absolute binding affinity prediction.

As a next step in our evaluation, we repeated the training of Pafnucy and GenScore using our PDBbind CleanSplit dataset. Strikingly, the performance of Pafnucy on the CASF2016 benchmark dropped substantially, approaching the level of the simple search algorithms (Fig. 3). GenScore proved to be more robust, as it suffered a much smaller relative drop in scoring performance when switching to PDBbind CleanSplit (Fig. 3). These performance drops support our hypothesis that the reported performance of many published binding affinity prediction models is boosted by data leakage.

### GEMS

**Fig. 4:**
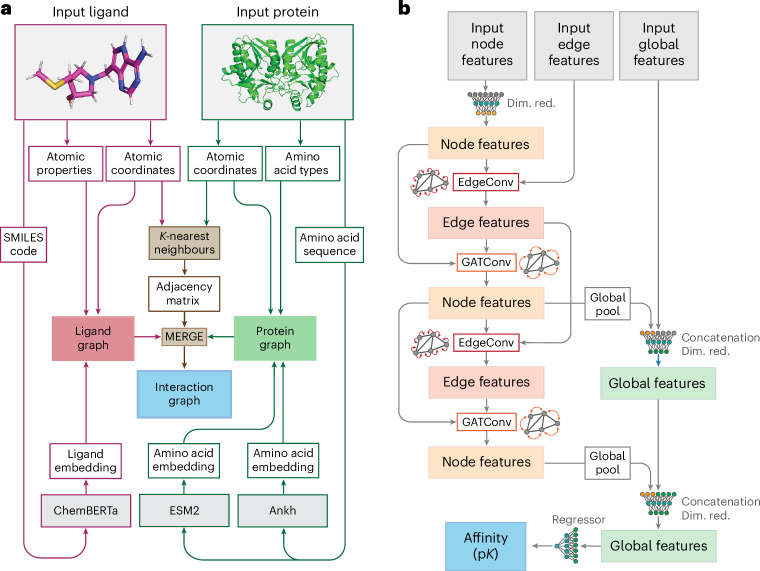
Graph-based modelling of protein–ligand interactions and GEMS model architecture. **a**, Schematic overview of the graph construction process used to model protein–ligand complexes in a sparse, rotation- and translation-invariant graph representation enhanced with language model embeddings. The core of these graph representations consists of an atom-level molecular graph of the ligand molecule (magenta) combined with an amino-acid-level graph of the protein pocket (green). During the merging of ligand and protein graphs, additional edges are introduced to connect ligand graph nodes (atoms) to protein graph nodes (amino acids) based on spatial proximity between ligand atoms and the atoms of the amino acids (interaction distance of 5 Å), computed using a *K*-nearest neighbours algorithm. The amino acid nodes are featurized with their type and embeddings derived from the protein language models ESM2 (ref. ^50^) and Ankh^51^. The ligand graph is featurized with atomic properties. The global features are intialized with a ligand embedding from the language model ChemBERTa-2 (ref. ^52^). **b**, GEMS model architecture for processing interaction graphs composed of node features, edge features and global context features. After an initial node feature dimensionality reduction (Dim. red.), node and edge features are transformed through an alternating sequence of node convolutions (GATConv) and edge convolutions (EdgeConv). The global graph features are dynamically updated throughout this process, incorporating pooled node representations after each node convolution. A final p*K* value prediction is made from the updated global features using a fully connected neural network.

Aiming to create a binding affinity prediction model that better generalizes to new data, we developed GEMS, a graph neural network that models protein–ligand structures as interaction graphs enhanced with embeddings from language models and processes these graphs through a series of graph convolutions to predict absolute binding affinities (Fig. 4).

When trained on PDBbind, GEMS showed a benchmark performance comparable with those of the top deep-learning-based scoring functions reported to date. Training GEMS on PDBbind CleanSplit initially resulted in much lower benchmark performance, as would be expected when removing data leakage. However, after substantial architectural optimizations and the integration of language model embeddings to enrich the feature space of the graphs, our GEMS model achieved competitive results on the CASF2016 benchmark (Fig. 3). With a prediction r.m.s.e. of 1.308 and a Pearson correlation of 0.803, GEMS considerably outperforms Pafnucy (r.m.s.e. = 1.484, Pearson *R* = 0.746) and GenScore (r.m.s.e. = 1.362, Pearson *R* = 0.780) when trained on PDBbind CleanSplit (Fig. 3). Moreover, GEMS even surpasses the reported performance metrics of several deep-learning-based scoring functions that were trained on the full PDBbind dataset and therefore benefitted from substantial train–test data leakage (Table 1).

**Extended Data Fig. 1:**
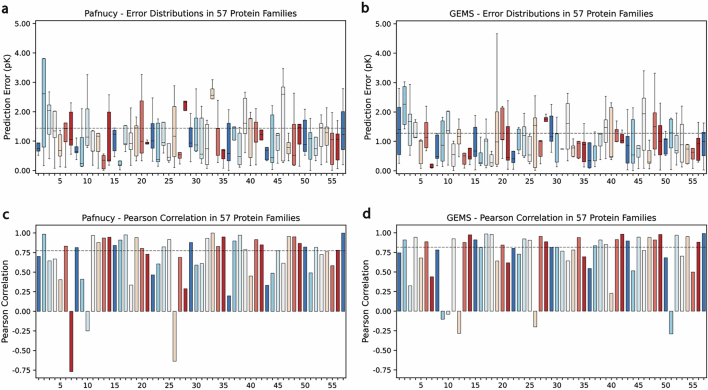
Performance of Pafnucy and GEMS in Different Protein Families. Comparison of the prediction accuracy of Pafnucy and GEMS per protein cluster in the CASF2016 benchmark dataset (N=285). **a**) and **b**) Distribution of absolute prediction errors (pK unit) of Pafnucy and GEMS within 57 protein clusters. Each box represents a cluster of five protein-ligand complexes of the same protein family with ligands spanning a wide range of binding affinities (n=5, unit=protein-ligand complexes). The black dashed lines indicate the prediction RMSE on the entire CASF2016 dataset. **c**) and **d**) Prediction Pearson correlations of Pafnucy and GEMS within the same 57 protein clusters. Black dashed lines indicate the Pearson correlation on the entire CASF dataset. Boxplots show the median (centre line), 25th-75th percentiles (box), whiskers extend to data points within 1.5 × IQR. Source data

In addition to its scoring power, we also evaluated GEMS’ accuracy and ranking power in different protein families. The ranking power of a scoring function refers to its ability to accurately rank ligands for a given target protein based on their binding affinities. The CASF2016 dataset features 57 clusters of distinct protein families, each consisting of five identical proteins paired with diverse ligands that span a wide range of binding affinities^10^. When trained on PDBbind CleanSplit, GEMS outperformed both Pafnucy and GenScore, as underscored by the distribution of Spearman correlation coefficients across the 57 clusters (Fig. 3c) and the favourable absolute error distributions across most clusters (Extended Data Fig. 1). To provide additional evidence of generalization, we evaluated GEMS on a challenging out-of-distribution (OOD) benchmark dataset proposed by Valsson et al.^46^, achieving competitive results (Supplementary Fig. 4).

#### Ablation

**Fig. 5:**
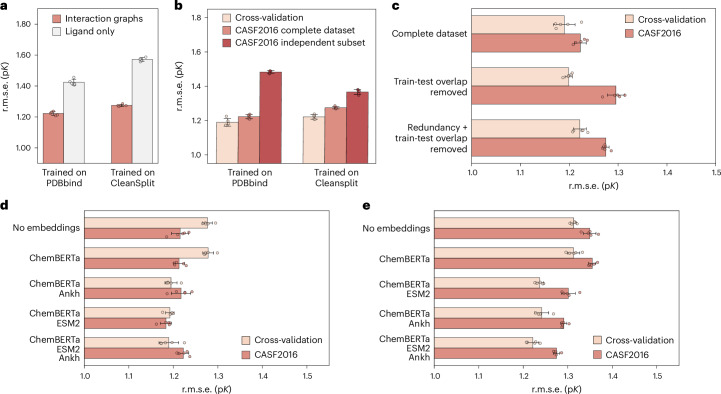
Impact of ablation and dataset filtering on GEMS performance on CASF2016 (*n* = 285). **a**, Ablation study showing the impact of removing all protein information from the graphs on CASF2016 performance, comparing GEMS models trained on PDBbind (left) and PDBbind CleanSplit (right). **b**, Comparison of GEMS performance on cross-validation, CASF2016 and the independent subset of CASF2016 (*n* = 144) for models trained on PDBbind (left) and PDBbind CleanSplit (right). **c**, CASF2016 performance of GEMS with varying levels of training dataset filtering: complete dataset (PDBbind), train–test overlap removed and both overlap and redundancy removed (PDBbind CleanSplit). **d**, Effect of incorporating language model embeddings on the cross-validation and CASF2016 performance of GEMS models trained on the original PDBbind dataset. **e**, Effect of incorporating language model embeddings on the cross-validation and CASF2016 performance of GEMS models trained on the PDBbind CleanSplit. All bars represent mean performance values of five models trained with five-fold cross-validation at different random seeds (*n* = 5, unit = independently trained models), with dots showing the individual performance values. Error bars represent data uncertainty, calculated as the standard deviation of the performance across the five models. Source data

**Extended Data Fig. 2:**
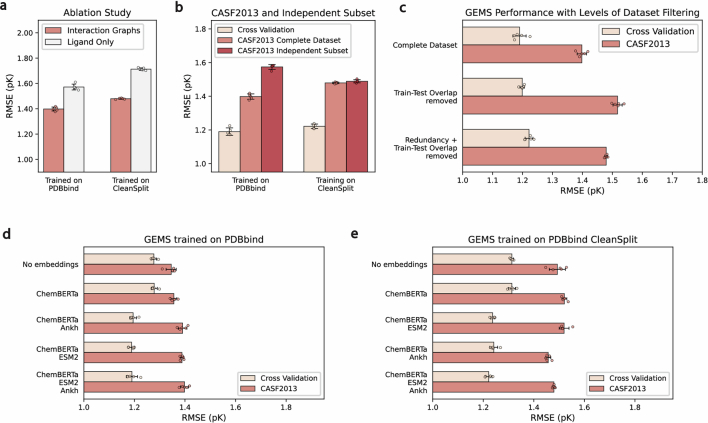
Impact of Ablation and Dataset Filtering on GEMS Performance on CASF2013 (N=195). **a**) Ablation study showing the impact of removing all protein information from the graphs on CASF2013 performance, comparing GEMS models trained on PDBbind (left) and PDBbind CleanSplit (right). **b**) Comparison of GEMS performance on cross-validation (CV), CASF2013, and the independent subset of CASF2013 (N=101), for models trained on PDBbind (left) and PDBbind CleanSplit (right). **c**) CASF2013 performance of GEMS with varying levels of training dataset filtering: complete dataset (PDBbind), train-test overlap removed, and both overlap and redundancy removed (PDBbind CleanSplit). **d**) Effect of incorporating language model embeddings on the cross-validation and CASF2013 performance of GEMS models trained on the original PDBbind dataset. **e**) Effect of incorporating language model embeddings on the cross-validation and CASF2013 performance of GEMS models trained on the PDBbind CleanSplit. All bars represent mean performance values of five models trained with 5-fold cross-validation at different random seeds (n = 5, unit=independently trained models), with dots showing the individual performance values. Error bars represent data uncertainty, calculated as the standard deviation of the performance across the five models. Source data

When trained on the original and unfiltered PDBbind, all tested GEMS model variants achieved competitive CASF2016 performance even after removing all protein information from the input data (r.m.s.e. = 1.424) (Fig. 5a and Extended Data Fig. 2a). According to this evaluation, these models—when trained only on ligands—make more accurate predictions than the scoring function of AutoDock Vina and even outperform several published deep-learning-based scoring functions. These results align with other studies indicating that models trained on PDBbind can achieve remarkably high performance on the CASF benchmark even when one interaction partner is deleted from the input data^35,36,42,43^. As these models received no protein information, their predictions are clearly not based on an understanding of protein–ligand interactions.

By contrast, when GEMS was trained on PDBbind CleanSplit, it produced very inaccurate benchmark predictions after protein nodes were omitted from the data (r.m.s.e. = 1.572). This notable performance drop indicates that when data leakage and redundancies are reduced, models must rely on an understanding of protein–ligand interactions to make accurate predictions.

#### Generalization to independent subset of CASF dataset

To explore whether models trained on PDBbind CleanSplit can generalize better to unseen complexes, we evaluated the performance of our models on a subset of the CASF2016 benchmark dataset, which was independent even before filtering the training data. According to the stringent similarity thresholds of our filtering algorithm, a fraction of the CASF2016 test dataset (144/285 complexes) is independent, with no similar complexes present in PDBbind. This independent subset thus provides a more reliable measure of generalization capability for models trained on PDBbind. Although GEMS trained on PDBbind showed high overall performance on the complete CASF2016 dataset (r.m.s.e. = 1.223), the performance on the independent subset was much lower (r.m.s.e. = 1.483). Notably, when tested on the same independent subset, the GEMS model trained on PDBbind CleanSplit performed better than the model trained on PDBbind (r.m.s.e. = 1.367) despite the substantial training dataset size reduction, suggesting that the true generalization capability of the model trained on PDBbind CleanSplit is indeed superior (Fig. 5b and Extended Data Fig. 2b).

#### Influence of training redundancy

Our filtering approach for compiling PDBbind CleanSplit not only reduced train–test overlap but also training dataset redundancy. Naturally, the reduction of the train–test overlap led to the anticipated drop in test set performance compared with models trained on the complete PDBbind dataset (Fig. 5c and Extended Data Fig. 2c). Reducing training dataset redundancy had two interesting effects: (1) it caused a decrease in cross-validation performance, supporting our hypothesis that these redundancies had previously inflated validation results; (2) we observed a consistent improvement in test set performance (Fig. 5c and Extended Data Fig. 2c). This is surprising, as the redundancy removal process excluded a substantial part of the training dataset (*N* = 1,451), which would conventionally be anticipated to reduce model performance. The observed positive effect suggests that extensive redundancies can be problematic in affinity prediction models, as models are prone to overfit to these clusters to minimize the training loss. Such overfitting interferes with learning the causal relationships underlying binding affinity and leads to models with lower generalization. In this case, distilling the training data into a smaller but more diverse collection of the most relevant complexes can be helpful for model training.

#### Influence of language model embeddings

Models trained on PDBbind and PDBbind CleanSplit exhibit a distinct response to the incorporation of language model embeddings (Fig. 5d,e and Extended Data Fig. 2d,e). As these embeddings are rich in biological and chemical information, initializing graph features with them is expected to improve performance in the challenging task of binding affinity prediction. When training on PDBbind, the GEMS baseline model, which lacks any language model embeddings, performed best on the CASF2016 test dataset. Incorporating language model features resulted in a continuous increase in cross-validation performance, without a corresponding improvement in test set performance. Conversely, when trained on PDBbind CleanSplit, the baseline model without language model features showed relatively low cross-validation and test dataset performance. However, the introduction of such features led to simultaneous improvements in both metrics. This suggests that PDBbind CleanSplit provides a better foundation for training protein–ligand affinity prediction models, as it eliminates straightforward paths to high test performance, such as exploiting biases and data leakages. Instead, the diversity inherent in the filtered dataset and the absence of structural similarities requires models to approach the task by actually learning the factors driving high-affinity protein–ligand interactions. These models benefit from increased model complexity and enrichment of features with language model embeddings.

## Discussion

The PDBbind dataset remains the largest resource for training protein–ligand binding affinity prediction models. However, the development of a generalizable affinity prediction model requires refining this dataset to address its substantial training redundancies and data leakage into the commonly used CASF benchmark. By developing a structure-based filtering algorithm, we created PDBbind CleanSplit, a refined training dataset with minimized redundancy and strict separation from the CASF complexes. With PDBbind CleanSplit, models can no longer rely on training data memorization, as all complexes resembling any from the CASF benchmarks have been excluded from the training dataset. In addition, the removal of redundancy ensures that models are trained on a much more diverse dataset, ultimately improving their generalization capabilities. In summary, PDBbind CleanSplit provides an improved foundation for training binding affinity prediction models, setting a new standard for robust training and reliable evaluation in this field.

The impact of using PDBbind CleanSplit for training becomes evident in the performance drops of Pafnucy and GenScore, revealing that the true generalization capability of these previously top-performing model is much lower than reported. By contrast, our GEMS scoring function maintained high prediction accuracy when trained on PDBbind CleanSplit, achieving performance comparable with many deep-learning-based binding affinity prediction models that trained on the original PDBbind and profited from the associated data leakage. In addition, training GEMS was about 25 times faster than training Pafnucy and over 100 times faster than GenScore on the same GPU, thanks to our sparse graph-based modelling of protein–ligand interactions and an efficient GNN architecture. Combined with transfer learning from large language models, GEMS obtained an understanding of protein–ligand interactions and thus can better generalize to strictly external test datasets.

GEMS is a powerful scoring function designed to address a critical bottleneck in SBDD. Although recent generative models such as AlphaFold3, RFdiffusion and DiffSBDD can create libraries of new protein–ligand interactions, their impact is constrained by the lack of tools to accurately predict binding affinities. Importantly, scoring de novo protein–ligand interactions demands models that go beyond exploiting structural similarities to existing complexes and demonstrate true generalization. GEMS fills this gap by offering robust scoring capabilities validated on strictly independent datasets, enabling identification of interactions with therapeutic potential.

We have prioritized accessibility by making our data, code and model publicly available, including datasets of precomputed interaction graphs for fast reproduction of our results and scripts for filtering the PDBbind database based on precomputed pairwise similarity matrices.

Promising future directions to further fine tune PDBbind CleanSplit include the incorporation of more sophisticated methods for ligand similarity computation. Although the current alignment and r.m.s.e.-based strategy employed in the filtering algorithm is effective at reducing data leakage and efficient enough to compute the 189 × 10^6^ pairwise similarities in PDBbind, its precision could be further improved using more advanced ligand comparison techniques, such as ROCS similarity scores^47^. Moreover, exploring more complex graph convolutional modules could provide valuable insights and represent a potential avenue for improvement.

The current architecture and training regime of GEMS were specifically optimized to achieve state-of-the-art absolute binding affinity prediction on structurally diverse protein–ligand complexes in a data-leakage-free setting. For this reason, GEMS was trained on a dataset of high-quality structures with reduced redundancy, and it has not been exposed to docking poses or decoy conformations during training. As a result of this design, we recommend using classical scoring functions or machine learning models that have been trained on appropriately augmented datasets for tasks requiring fine discrimination between closely related docking poses, such as selecting the best conformation from many Glide or AutoDock Vina outputs. One avenue to improve GEMS in this direction is to augment its training data with high-quality docking poses and explicitly train the model to distinguish and rank closely related binding conformations. This would enhance the applicability of GEMS to docking-based virtual screening scenarios, where pose selection among many near-native docking conformations is usually required. Other desirable improvements include the ability to provide atom-level importance scores or optimize molecular conformations directly^48^.

## Methods

### Datasets

**Extended Data Fig. 3:**
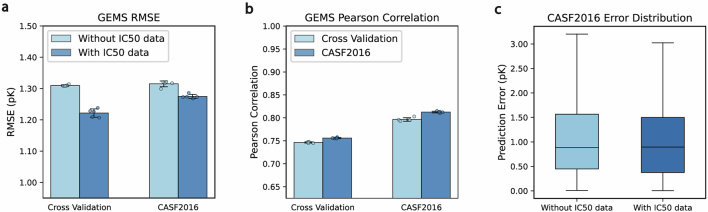
Performance of GEMS with and without IC50-labeled complexes. Comparison of cross validation and CASF2016 (N=285) prediction accuracy of GEMS in terms of **a**) RMSE, **b**) Pearson correlation and **c**) absolute prediction errors on CASF2016 depending on inclusion of 6400 complexes labeled with IC50 values in the training data. The bars in **a**) and **b**) represent the mean performance values of five models trained with 5-fold cross-validation at different random seeds (n = 5, unit=independently trained models), with dots showing the individual performance values. Error bars represent data uncertainty, calculated as the standard deviation of the performance across the five models. To compute the distributions of absolute prediction errors in **c**), the predictions of the five models were averaged into an ensemble prediction for each of the 285 CASF2016 complexes (n=285, unit=protein-ligand complexes). Boxplots show the median (centre line), 25th-75th percentiles (box), whiskers extend to data points within 1.5 × IQR. Source data

The main data resource used in this work was the PDBbind (v.2020) database, containing 19,443 protein–ligand complexes from the Protein Data Bank (PDB) with experimentally measured binding affinities. This database is split into a general set (*n* = 14,127) and a refined set (*n* = 5,316) that has been compiled based on strict curation criteria, including crystallographic structures (excluding NMR structures) with a resolution of <2.5 Å and an inhibition constant (*K*_i_) or dissociation constant (*K*_d_) in the range of 1 pM to 10 mM (p*K* range 2–12). To keep our training set as large as possible, we used a merged dataset containing all data from the general and the refined set as training data, excluding all complexes present in the CASF benchmark datasets (versions 2013 and 2016), which served as external test datasets in this work. Each complex was labelled with either an inhibition constant *K*_i_, the dissociation constant *K*_d_ or the half-maximal inhibitory concentration IC50. Despite concerns about the comparability of IC50 with *K*_i_/*K*_d_ measurements^49^, these metrics were considered interchangeable in this study and converted to p*K* values with $-{\log }_{10}(K_{\rm{i}}/K_{\rm{d}}/\rm{IC}50)$ to generate the final affinity labels. This decision is based on the observation that including IC50 complexes (*n* = 6,400) in the training data, despite being more noisy, has a small positive effect on the prediction performance of models on an independent test dataset (Extended Data Fig. 3), confirming the findings of previous research^24^.

During data preprocessing and graph construction, some protein–ligand complexes were excluded from the datasets based on the following criteria.

- Affinity label is not exact; for example, *K*_i_ < 100 nm (*n* = 383)
- Error in RDKit when handling explicit hydrogens in some SDF files (*n* = 45)
- Protein contains unknown residue or heteroatom in binding pocket, such as UNK or DOD (*n* = 14)
- Error occurring during RDKit parsing due to incorrect valences in SDF files (*n* = 5)
- Protein structure is not completely resolved and atoms are missing from the binding pocket (*n* = 2)
- The ligand contains fewer than five heavy atoms (*n* = 1)

This filtering reduced the size of the training dataset by 450 complexes to *N* = 18,623 protein–ligand complexes. The CASF2016 (*N* = 285) and CASF2013 (*N* = 195) test sets were unaffected.

### Filtering algorithm

To identify and remove structural similarities between the PDBbind database and the CASF benchmark datasets, our dataset filtering process used a combination of Tanimoto ligand similarity, protein similarity (TM scores) and a pocket-aligned ligand r.m.s.d. to compare the positioning of the ligands within the protein pockets. Tanimoto similarity is commonly used in cheminformatics to measure the similarity between small molecules^45^. Based on comparing the chemical fingerprints, this score ranges between 0 (no similarity) and 1 (identical) and is a useful metric to identify compounds with similar structural and chemical properties. This score served as the first layer of our filtering, identifying pairs of complexes with similar ligands. The second layer of the filtering process used TM-align^44^, a computational tool designed to compare protein structures by finding the optimal alignment of their three-dimensional shapes. It uses a scoring function based on the r.m.s. distance of aligned residues and a length normalization factor, making it particularly effective for identifying structural similarities between proteins, regardless of sequence similarity. In our application, TM-align was valuable for identifying proteins that share similar binding pockets despite having low sequence identity. For example, the test complex 1P1N and the training complex 3U92 share 53% sequence identity. Nevertheless, TM-align has recognized that 1P1N is well represented within 3U92 and returns a TM score of 0.93. Alignment of the proteins then revealed that these complexes share identical binding pockets (Supplementary Fig. 1). The capability of our filtering process to identify complexes with similar interaction patterns in proteins that are otherwise dissimilar is a key advantage of our method over traditional sequence-based clustering approaches.

However, the combination of TM score and Tanimoto similarity does not conclusively determine the similarity of the interaction patterns of two complexes. Even with a Tanimoto similarity of 1 and a TM score of 1, two complexes might have different positioning of the ligand within the binding pocket, or the ligands might even bind at entirely different sites of the protein. Therefore, the third layer of our filtering process compares ligand positioning through a protein alignment followed by an r.m.s.d. calculation between the ligand atoms. For this analysis, one ligand’s atom coordinates are translated and rotated using the rotation matrix and translation vector obtained from TM-align (Supplementary Fig. 2). This alignment positions both ligands within the coordinate system of the optimal alignment of the protein structures. The r.m.s.d. between the ligand atoms is then calculated to provide a quantitative measure of the positional similarity.

Our filtering algorithm imposes stringent rules on the structural and chemical similarity within the dataset. The similarity between two complexes is quantified on the basis of four computed similarity metrics.

- *Affinity*: The absolute difference between the reported binding affinities (p*K* values) was calculated.
- *Tanimoto*: The Tanimoto similarity score to compare ligand structure was computed using RDKit library v.2024.03.3 based on count-based molecular fingerprints (GetCountFingerprint) of size 2048 and radius 2.
- *TM score*: TM-align^44^ was used to assess protein structural similarity and to align complexes based on their protein residues. This algorithm identifies the best structural alignment between protein pairs and outputs a TM score ranging from 0 (dissimilar structures) to 1 (identical structures) along with the translation vector and rotation matrix necessary to achieve optimal alignment. To find whether one of the proteins is well represented within the other, we considered TM scores normalized by the lengths of both amino acid chains and used the highest result as a similarity score.
- *r.m.s.d.:* An r.m.s.d. was computed to compare ligand positioning in the binding pocket of the proteins. For this, the protein structures were aligned together with the bound ligands by applying the translation vector and rotation matrix that TM-align used to generate an optimal protein alignment. Then the atom coordinates of the aligned ligands were compared by computing the r.m.s.d. between the nearest points in the two point clouds. In case of different atom counts, the distances from each atom in the larger ligand to its nearest neighbour in the smaller ligand were used, resulting in larger r.m.s.d. values for ligands with different atom counts. Given two complexes with similar ligands, lower values indicate a very similar positioning of the two ligands in their binding pockets, and a higher value suggests very dissimilar binding conformations (or even binding at a different location of the protein).

#### Removal of train–test overlap

To remove the overlap between the training dataset (PDBbind) and the CASF test datasets (CASF2013 and CASF2016), our filtering algorithm removed all training complexes that were similar to any test complex in terms of protein structure, ligand structure, ligand binding and affinity. This eliminated data leakage by removing shared similarities between training and test datasets, effectively bringing the CASF datasets closer to being independent test datasets. A training complex was excluded if it shared all of the following similarities with a test complex.

- The proteins had a TM score higher than 0.8.
- The sum of the Tanimoto score (*T*) and the inverted r.m.s.d. was higher than 0.8: (*T* + (1 − r.m.s.d.) > 0.8).
- The affinity labels were similar (±1 in p*K* units).

**Extended Data Fig. 4:**
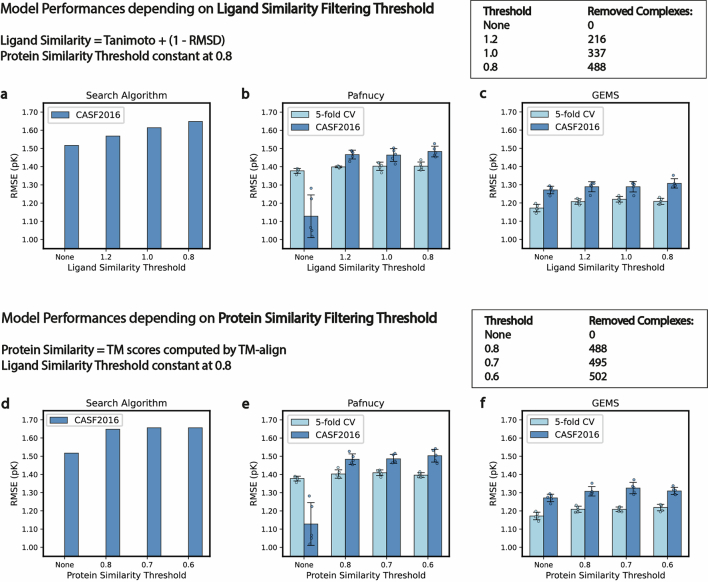
Influcence of Filtering Thresholds on Model Performance. Changes in cross-validation and CASF2016 (N=285) prediction accuracy of the complex-based search algorithm, Pafnucy and GEMS in dependence of different training dataset filtering thresholds for protein similarity and ligand similarity. **a**), **b**) and **c**): Changes in model performance with varying ligand similarity thresholds at constant protein similarity threshold 0.8. **d**), **e**) and **f**) Changes in model performance with varying protein similarity thresholds at constant ligand similarity threshold 0.8. Ligand similarity reflects a combined assessment of Tanimoto scores and pocket-aligned ligand RMSD (Tanimoto + 1-RMSD). Protein similarity represents TM-scores computed by TM-align. The first group of bars in each plot represents the model performance without any training dataset filtering. The performance values in **b**), **c**), **e**) and **f**) reflect the mean validation and CASF2016 RMSE of five models originating from 5-fold cross-validation (n=5, unit=independently trained models), with dots showing individual performance values and error bars representing the standard deviation across the performance of the five models. To ensure a fair comparison, Pafnucy and GEMS were trained using the exact same 5-fold cross-validation split. Source data

The first criterion ensured that training complexes were only excluded if they shared a high structural similarity with a test complex. The second criterion balanced ligand chemical similarity with binding conformation similarity. This approach means that a greater ligand positioning similarity is acceptable for two ligands with modest chemical similarity (for example, Tanimoto coefficient, *T* = 0.6). Conversely, for very similar ligands (*T* approaching 1), the training complex could be excluded from the dataset despite larger deviations in positioning. The third criterion ensured that training complexes were only excluded if they shared a similar affinity label with the test complex. In addition, training complexes were excluded if they had an identical ligand (Tanimoto > 0.9) and a closely matching affinity label (±1) compared with a test complex. Together, this filtering excluded 711 training complexes. An overview over the model performance of GEMS, Pafnucy and the complex-based search algorithm in dependence of different dataset filtering thresholds for protein similarity and ligand similarity can be found in Extended Data Fig. 4.

#### Removal of training dataset redundancy

A second filtering layer was applied to the training dataset to eliminate excessive redundancies while preserving the largest possible dataset size and quality. Initially, pairwise similarities between all training complexes were calculated using the described similarity metrics and recorded in a pairwise similarity matrix. To identify clusters of high similarity, this matrix was transformed into an adjacency matrix by applying the following thresholds.

- The proteins had a TM score higher than 0.8.
- The sum of the Taminoto score (*T*) and the inverted r.m.s.d. was higher than 1.3: *T* + (1 − r.m.s.d.) > 1.3.
- The affinity labels differed by less than ±0.5 in p*K* units.

The resulting adjacency matrix connected all data points that met these criteria. The filtering algorithm then iteratively removed complexes from the training dataset until no connections remained in the adjacency matrix. During each iteration, the complex with the highest number of connections (indicating the most similarities with other complexes) was excluded. In cases where multiple complexes had the same number of connections, preference was given to removing those from the PDBbind general set rather than the refined set. If ties persisted, the complex with the lowest resolution was preferentially excluded. In total, this filtering process excluded 1,451 training complexes and allowed us to confidently train models using five-fold cross-validation with random splitting, without the risk of inflated validation performance that could arise from similarities between training and validation datasets.

### Graph construction

Each complex in the PDBbind database was translated into an affinity-labelled graph that models the interaction between the ligand and the protein (hereafter referred to as interaction graphs). For this, the affinity labels of all protein–ligand complexes in the database were extracted from the index files provided by the PDBbind database. The supplied inhibition constants (*K*_i_), dissociation constant (*K*_d_) and half-maximal inhibitory concentration (IC50) were converted to p*K* values with $-{\log }_{10}(K_{\rm{i}}/K_{\rm{d}}/\rm{IC}50)$ to generate the final affinity labels.

The interaction graphs were generated as follows. Protein PDB files were processed with Biopython v.1.83. Ligand SDF files were parsed with RDKit v.2024.03.3. From the atom coordinates of the ligand and protein, a pairwise distance matrix was generated to identify ligand atoms and protein atoms in close vicinity. An interaction distance of 5 Å was considered sufficient to capture the most critical molecular interaction between ligand and protein. Consequently, if any atom of a protein residue was closer to a ligand atom than 5 Å, the protein residue was considered to be in interaction distance to this ligand atom. To generate a graph representation of the protein–ligand interaction, a basic molecular graph was generated from the ligand by translating the atoms into graph nodes and the covalent bonds into graph edges. To add an encoding of the protein pocket to these ligand graphs and model potential non-covalent interactions, all protein residues and heteroatoms previously determined to be in interaction distance were added as additional graph nodes and connected to all ligand atoms within interaction distance through additional edges. Importantly, the protein was represented at the amino acid level, which meant that each amino acid was represented by a single graph node located at the position of its Cα atom (Supplementary Fig. 3). All edges of these basic interaction graphs were undirected (message-passing in both directions) and self-loops were included for each node.

#### Featurization

The interaction graphs’ initial node and edge features consisted of one-hot-encoded chemical properties computed with the RDKit v.2024.03.3 library. The following basic chemical features were included.

- Nodes: atom type (B, C, N, O, P, S, Se, metal, halogen), ring membership, hybridization, formal charge, aromaticity, atomic mass, number of bonded hydrogens, degree, chirality
- Edges: edge type (covalent, self-loop, non-covalent), length, bond type (single/double/triple/aromatic), conjugation, ring membership, stereochemistry

In feature vectors of edges connecting a ligand atom node with a protein residue node, the length feature was replaced with four values representing the distances between the ligand atom and the four main backbone atoms of the residue (N, Cα, C and virtual Cβ). If any feature did not apply to a certain node (for example, atom type for a node representing a protein residue), the feature was replaced with zero padding to maintain consistent feature dimensions across the graphs.

The features of the nodes representing protein residues were additionally supplemented with a vector containing the one-hot-encoded amino acid type and with amino acid embeddings. These embeddings were generated with ESM2 (T6 8M checkpoint downloaded from huggingface https://huggingface.co/facebook through the transfomers library v.4.33.3)^50^ and ANKH (base model downloaded through the ankh python library v.1.10.0)^51^. For this, the amino acid sequence of a protein was passed through the downloaded tokenizers and model checkpoints. The resulting matrices contained embeddings for each amino acid in the protein sequences, which were then appended to the features of the corresponding graph nodes.

In addition, a ligand embedding was computed for each complex using the ChemBERTa-2 language model (ChemBERTa-77M-MLM downloaded from https://huggingface.co/DeepChem through the transformers library v.4.33.3) (ref. ^52^). The SMILES codes of all ligands in the dataset were generated with RDKit v.2024.03.3 and passed through the downloaded model to obtain ligand embeddings. These embeddings were used to initialize the graph’s initial global features.

### Model architecture and training

Our GNN models were implemented using PyTorch v.2.0.1 and Torch Geometric (pyg) v.2.5.2. The model architecture captures and integrates multilevel graph information across nodes, edges and the entire graph. It receives batches of interaction graphs as input and alternates between updating the graph’s edge features, node features and global features (Fig. 4b). Finally, the global features are passed through a dropout layer and two fully connected layers with a ReLU activation to produce the final output. The entire model architecture of GEMS encompasses five convolutional layers, of which three update the node features and two update the edge features, resulting in a model architecture with 1,032,129 learnable parameters.

As graph convolutional operator implemented in the NodeModel module, GATv2Conv^53^ from Torch Geometric was selected with concatenation of multihead attention. This convolutional layer computes updated node features ${x}_{i}^{{\prime} }$ for node *i* with

1 $${{\bf{x}}}_{i}^{{\prime} }={\alpha }_{i,i}{\varTheta }_{\rm{s}}{{\bf{x}}}_{i}+\sum _{j\in {\mathcal{N}}(i)}{\alpha }_{i,\,j}{\varTheta }_{\rm{t}}{{\bf{x}}}_{j},$$

where **x**_*j*_ represents the feature vector of the neighbouring node *j*, ${\mathcal{N}}(i)$ is the set of neighbouring nodes of node *i*, *Θ*_s_ is a learnable weight matrix applied to the features of the source node *i*, *Θ*_t_ is a learnable weight matrix applied to the features of the target node *j* and *α*_*i*, *j*_ are the attention coefficients representing the importance of node *j*’s features to node *i*. The attention coefficients *α*_*i*, *j*_ are computed as

2 $${\alpha }_{i,\,j}=\frac{\exp \left({{\bf{a}}}^{\top }{\rm{LeakyReLU}}\left({\varTheta }_{\rm{s}}{{\bf{x}}}_{i}+{\varTheta }_{\rm{t}}{{\bf{x}}}_{j}\right)\right)}{{\sum }_{k\in {\mathcal{N}}(i)\cup \{i\}}\exp \left({{\bf{a}}}^{\top }{\rm{LeakyReLU}}\left({\varTheta }_{\rm{s}}{{\bf{x}}}_{i}+{\varTheta }_{\rm{t}}{{\bf{x}}}_{k}\right)\right)}.$$

where LeakyReLU is an activation function applied to introduce nonlinearity. Taken together, this layer updates each node’s features by aggregating the transformed features of its neighbours and itself, weighted by attention coefficients that are dynamically computed based on the features of both the source and target nodes. This mechanism allows the model to focus on the most relevant parts of the graph structure during learning.

#### Model training

During the training of GEMS, all model variants trained on the same dataset were subjected to the same five-fold cross-validation split to eliminate the variability introduced by differences in data partitioning. The models were trained across all five splits and the models that achieved the lowest validation r.m.s.e. were saved. The training objective was to minimize the r.m.s.e. of the predicted p*K* values of the training complexes, using a stochastic gradient descent optimizer. To prevent overfitting, early stopping was implemented, halting the training process if validation r.m.s.e. did not improve for 100 consecutive epochs. All models were trained on one NVIDIA GeForce RTX 4090 or one NVIDIA GeForce RTX 3090 for approximately 200–1,200 epochs (depending on the early stopping), taking between 10 and 60 min.

#### Model selection

To robustly determine the uncertainty of GEMS and select the best model variant, we trained all variants using our five-fold cross-validation approach at five different random seeds. This approach ensured that the randomness in the data splitting differs with each training run, which allowed us to estimate the variability in performance that arises from different data splits. By averaging the outcomes and calculating the standard deviation between these iterations, we generated error bars for our performance metrics.

From all trained models, the one with the highest and most consistent validation performance across all five folds was selected. This ensured that the model with the most robust generalization across different subsets of our training data was selected. For testing models on the CASF test datasets, the CASF complexes were passed through all five cross-validation models and the predictions from all five models were averaged to generate the final ensemble predictions.

#### Ablation

To test whether GEMS relies on the presence of both ligand and protein data, we generated ligand-only versions of the PDBbind, PDBbind CleanSplit and CASF datasets by removing all protein nodes, leaving only the molecular graph of the ligand. We then trained and tested GEMS as described above using these ligand-only datasets.

#### Pafnucy

The Pafnucy model was obtained from the authors public repository (https://gitlab.com/cheminfIBB/pafnucy) and trained on a NVIDIA GeForce RTX 3090 GPU using the provided scripts and instructions. Protein–ligand complexes were preprocessed and datasets prepared according to the authors’ specifications. We evaluated its performance on the training and validation sets, as well as on the CASF2016 dataset, using the predictions from the output text file generated by the training script. To compare the performance of our model with that of Pafnucy, we used the same cross-validation method and identical data split.

#### GenScore

The GenScore model was obtained from the authors public repository (https://github.com/sc8668/GenScore) and preprocessed graph datasets covering the full PDBbind database (v.2020) and the CASF2016 benchmark were downloaded from Zenodo (https://zenodo.org/records/7578480). Using these datasets, we trained and evaluated GenScore by running the provided training and inference scripts with default settings on a NVIDIA GeForce RTX 3090 GPU. To assess GenScore’s scoring power on CASF2016, we employed a benchmarking script from the official CASF2016 scoring power evaluation protocol^10^. As GenScore produces numerical scores that are not on the same scale as experimental binding affinities (p*K* values), the benchmarking script applies a simple linear regression to map the predicted scores to the experimental p*K* scale. From this regression model, we obtained the predicted binding affinity values for each complex, which allowed us to compute absolute prediction errors and r.m.s.e. values, as shown in Fig. 3. Because the provided GenScore code supports only random train–validation splits, we could not reproduce the exact data splits used in the GEMS and Pafnucy experiments. However, to enable the fairest possible comparison under these constraints, we trained five GenScore models using different random seeds and reported the mean performance and standard deviation across these runs. To train GenScore on the filtered PDBbind CleanSplit dataset, we filtered the provided training dataset to contain only the complexes in CleanSplit and repeated the above procedure.

#### Evaluation on the OOD benchmark dataset

To assess the generalization capability of GEMS, we evaluated the model on the OOD test dataset introduced by Valsson et al.^46^. Before evaluation, all complexes present in the OOD test set were removed from the training data to prevent data leakage. Subsequently, GEMS was trained with five-fold cross-validation and without additional hyperparameter optimization, before the OOD test dataset complexes were passed through the model to retrieve predicted p*K* values. For comparability with the results of Valsson et al., the experimental p*K* values and the predicted p*K* values were converted to binding free energies using the equation $\Delta G=-\ln (10)\times R\times T\times {\mathrm{p}}K$, where *R* = 1.987 × 10^−3^ kcal K^−1^ mol^−1^ is the universal gas constant and *T* = 297 K is the absolute temperature.

### Reporting summary

Further information on research design is available in the Nature Portfolio Reporting Summary linked to this article.

## Supplementary information

Supplementary Information Supplementary Note Architectural details and model selection, Supplementary Figs. 1–4, Supplementary Note Training behaviour, Supplementary Note Graph construction and Supplementary Note Model architecture.

Reporting Summary

## Source data

Source Data Fig. 3 *t*-SNE-reduced embedding and annotations for all structures in the three databases.

Source Data Fig. 5 Statistical source data for Fig. 3b,c.

Source Data Extended Data Fig. 1 Model performance on clusters depicted in Extended Data Fig. 1 (Pafnucy and GEMS).

Source Data Extended Data Fig. 2 Model performances depicted in Extended Data Fig. 2.

Source Data Extended Data Fig. 3 Model performance and predictions depicted in Extended Data Fig. 3.

Source Data Extended Data Fig. 4 Model performance depicted in Extended Data Fig. 4.

Source Data Table 1 Performances in Table 1 (search algorithms, Pafnucy and GenScore).

## Data Availability

All protein–ligand complex structures and binding affinity data used in this study were obtained from the PDBbind database^37,38^, which is publicly accessible at http://www.pdbbind.org.cn. The dataset is freely available to the research community and can be downloaded without restriction. The data generated in this study are freely available on GitHub (https://github.com/camlab-ethz/GEMS) including GEMS model parameters and PDBbind CleanSplit. For fast reproduction of our results, PyTorch datasets of precomputed interaction graphs and pairwise similarity matrices for the entire PDBbind database are available via Zenodo at 10.5281/zenodo.14260170 (ref. ^54^). Source data are provided with this paper.

## Code availability

The code generated in this study is freely available on GitHub. All scripts for dataset filtering, model training and inference can be accessed through https://github.com/camlab-ethz/GEMS. A docker container for easy reproduction and implementation is provided. For long-term accessibility and citation, the code is available via 10.5281/zenodo.15919357 (ref. ^55^).
