# Supplementary material for: Resolving data bias improves generalization in binding affinity prediction
Source: Nat Mach Intell. 2025 Oct 21;7(10):1713–25. doi: 10.1038/s42256-025-01124-5 (PMC12552120; doi:10.1038/s42256-025-01124-5)
Supplement: Supplementary file 1 — Supplementary Note Architectural details and model selection, Supplementary Figs. 1–4, Supplementary Note Training behaviour, Supplementary Note Graph construction and Supplementary Note Model architecture. [file 42256_2025_1124_MOESM1_ESM.pdf]

nature machine intelligence

Supplementary information https://doi.org/10.1038/s42256-025-01124-5

Resolving data bias improves generalization in binding affinity prediction

In the format provided by the authors and unedited

Supplementary Information

Supplementary Note - Architectural Details and Model Selection

GEMS was implemented using PyTorch v.2.0.1 and Torch Geometric (pyg) v.2.5.2. The model architecture captures and integrates multi-level graph information across nodes, edges, and the entire graph. It receives batches of interaction graphs as input and alternates between updating the graph's edge features, node features, and global features. The architecture includes the following components:

1. A NodeTransformMLP module, which applies a multi-layer perceptron (MLP) with ReLU and dropout to transform the input node features.
2. An EdgeModel module to update edge features. It processes the concatenated features of source nodes, destination nodes, and existing edge features through an MLP with ReLU and dropout.
3. A NodeModel module to update node features. It uses a graph attention network (GATv2Conv) convolution to update node features based on their neighboring nodes and edge attributes.
4. A GlobalModel module concatenates the global graph features with aggregated node features and applies an MLP with dropout to create updated global graph features.

The entire model architecture of GEMS encompasses five convolutional layers, of which three update the node features and two update the edge features, resulting in a model architecture with 1'032'129 learnable parameters. In a forward pass of the model, the node features are first transformed by the NodeTransformMLP module. These transformed node features, along with edge attributes and global features, are then processed through two consecutive graph layers. Each graph layer performs the following sequence of updates: the EdgeModel updates the edge features based on the connected node's features, the NodeModel updates the node features based on the features of neighboring nodes and the connecting edges, and the GlobalModel updates the global features with pooled node features. After the first graph layer, batch normalization is applied to the node, edge, and global features. Following the second graph layer, a dropout layer is applied to the global features to prevent overfitting. Finally, the global features are passed through two fully connected layers with a ReLU activation to produce the final output.

As graph convolutional operator implemented in the NodeModel module, GATv2Conv from Torch Geometric was selected with concatenation of multi-head attention. This convolutional layer computes updated node features x'_i for node i with

$$\mathbf{x}'_i = \alpha_{i,i}\boldsymbol{\Theta}_s\mathbf{x}_i + \sum_{j\in\mathcal{N}(i)} \alpha_{i,j}\boldsymbol{\Theta}_t\mathbf{x}_j, \tag{1}$$

where $\mathbf{x}_j$ represents the feature vector of the neighboring node j, $\mathcal{N}(i)$ the set of neighboring nodes of node i, $\boldsymbol{\Theta}_s$ a learnable weight matrix applied to the features of the source node i, $\boldsymbol{\Theta}_t$ a learnable weight matrix applied to the features of the target node j and $\alpha_{i,j}$ the attention coefficients representing the importance of node j's features to node i. The attention coefficients $\alpha_{i,j}$ are computed as

$$\alpha_{i,j} = \frac{\exp\left(\mathbf{a}^\top \mathrm{LeakyReLU}\left(\boldsymbol{\Theta}_s\mathbf{x}_i + \boldsymbol{\Theta}_t\mathbf{x}_j\right)\right)}{\sum_{k\in\mathcal{N}(i)\cup\{i\}} \exp\left(\mathbf{a}^\top \mathrm{LeakyReLU}\left(\boldsymbol{\Theta}_s\mathbf{x}_i + \boldsymbol{\Theta}_t\mathbf{x}_k\right)\right)}. \tag{2}$$

where LeakyReLU is an activation function applied to introduce non-linearity. Taken together, this layer updates each node's features by aggregating the transformed features of its neighbors and itself, weighted by attention coefficients that are dynamically computed based on the features of both the source and target nodes. This mechanism allows the model to focus on the most relevant parts of the graph structure during learning.

Hyperparameter Optimization: To find optimal hyperparameters, the following search space was tested:

- Number of message passing steps: Search space [1,2,3] with final value 2.
- Graph pooling operator: Search space [mean pooling, add pooling, max pooling] with final choice add pooling.
- NodeTransformMLP output channels: Search space [256, 128, 64] with final value 64.
- NodeModel output channels: Search space [256, 128, 64] with final value 64.
- EdgeModel output channels: Search space [128, 64] with final value 64.
- GlobalModel output channels: Search space [768, 512, 384, 256] with final value 384.

Model Training and Selection: During the training of GEMS and Pafnucy, all model variants trained on the same dataset were subjected to the same five-fold cross-validation split to eliminate the variability introduced by differences in data partitioning. The models were trained across all five splits, and the models that achieved the lowest validation root-mean-square-error (RMSE) were saved. The training objective was to minimize the RMSE of the predicted pK values of the training complexes, using a stochastic gradient descent (SGD) optimizer. To prevent overfitting, early stopping was implemented. This technique halted the training process if there was no improvement in the validation RMSE for 100 consecutive epochs. All models were trained on one NVIDIA GeForce RTX 4090 or one NVIDIA GeForce RTX 3090 for approximately 200-1200 epochs (depending on the early stopping), taking between 10 and 60 minutes. The training hyperparameters were optimized as follows:

- Batch size: Search space [128,256,512,640] with final value 256.
- Learning rate: Search space [0.0001, 0.001, 0.01] with final value 0.001.
- Weight decay: Search space [0.0001, 0.001, 0.01] with final value 0.001.

From all trained models, the one with the highest and most consistent validation performance across all five folds was selected. This ensures that the model with the most robust generalization across different subsets of our training data is selected. For testing models on the CASF test datasets, the CASF complexes were passed through all five cross-validation models and the predictions from all five models were averaged to generate the final ensemble predictions.

To robustly determine the uncertainty of GEMS, we trained it using our five-fold cross-validation approach at five different random seeds. This approach ensures that the randomness in the data splitting differs with each training run, which allows us to estimate the variability in performance that arises from different data splits. By averaging the outcomes and calculating the standard deviation between these iterations, we generated error bars for our performance metrics.

Supplementary Figure 1 - Detection of Similar Interaction Patterns Despite Low Sequence Identity

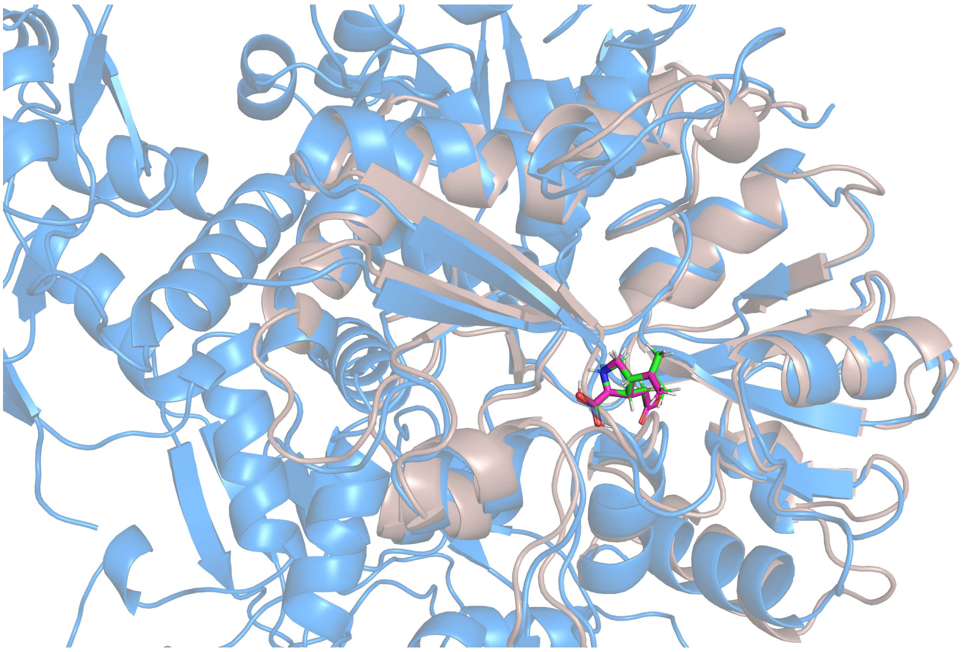

Supplementary Figure 1: Detection of Similar Interaction Patterns Despite Low Sequence Identity: Superposition of the test complex 1P1N (protein in gray, ligand in magenta) with the training complex 3U92 (protein in blue, ligand in green) structurally aligned with TM-align. These complexes have a low sequence identity of 53% but a high TM-score of 0.93, as 1P1N is a substructure of 3U92. They contain closely matching binding pockets, identical ligands, and similar binding conformations. Due to this substantial similarity, these complexes would provide nearly identical input data points to our models. Considering the comparable affinity labels, complex 3U92 was excluded from the training dataset to eliminate train-test data leakage. The capability to identify complexes with similar interaction patterns despite low sequence identity is a key advantage of our filtering algorithm over traditional sequence-based methods.

Supplementary Figure 2 - Pocket-Aligned Ligand RMSD Calculation

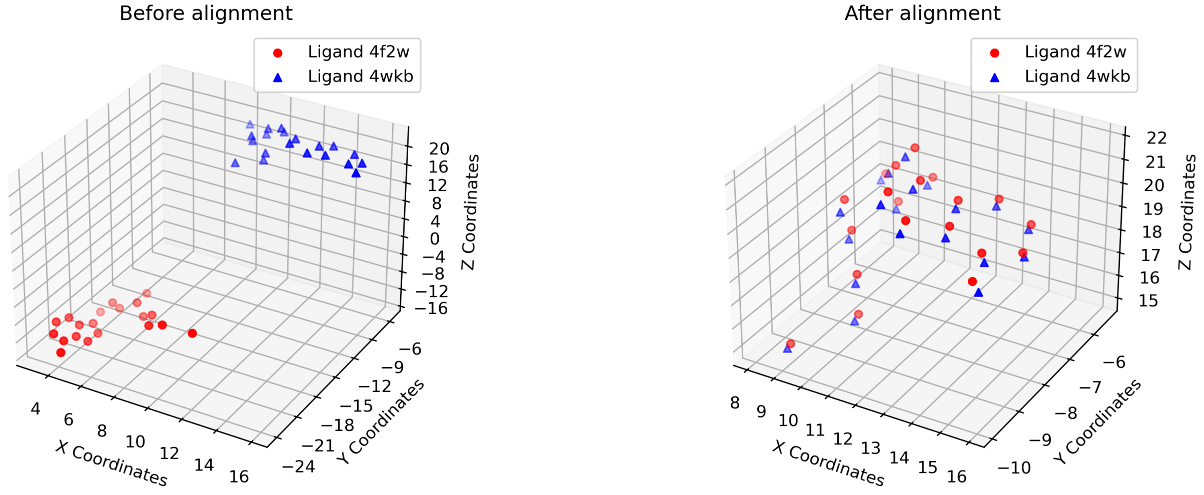

Supplementary Figure 2: Ligand Binding Conformation Similarity with Pocket-Aligned RMSD: a) Atom coordinates of the ligands of 4F2W and 4WKB before protein alignment. b) Atom coordinates of the ligands of 4F2W and 4WKB after protein alignment. The complexes 4F2W (CASF2016) and 4WKB (PDBbind) are highly similar, with a pK difference of 0.27, a Tanimoto score of 1.0 and a TM-score of 0.99, indicating identical ligands and nearly identical proteins. However, to conclusively assess the structural similarity of these complexes, it is necessary to compare the binding conformations of the ligands. For this, the complex 4F2W is translated and rotated into the coordinate system of the optimal protein alignment using the translation vector and rotation matrix returned by TM-align. This protein-alignment also aligns the contained ligands and reveals their nearly identical binding conformations (RMSD=0.33Å).

Supplementary Figure 3 - Interaction Graphs

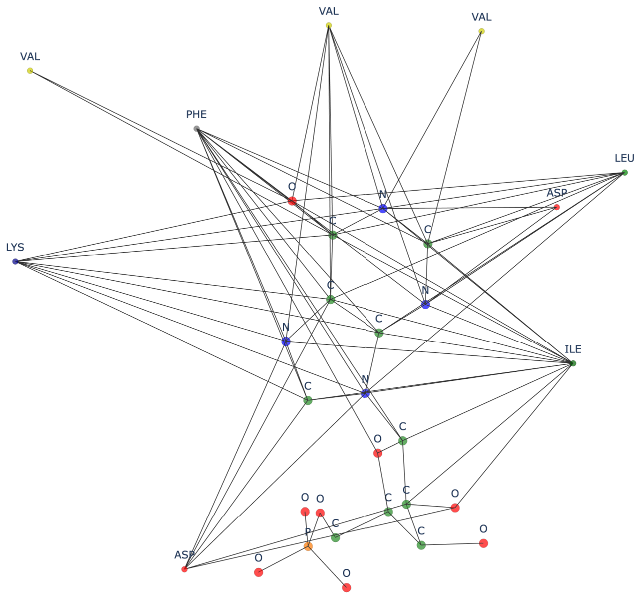

Supplementary Figure 3: Interaction Graph Generated From the 5kam Protein-Ligand-Complex: This example graph includes a graph representation of the ligand molecules, where atoms are represented as nodes and bonds as edges, and a residue-level representation of the protein pocket. Edges between ligand atoms represent covalent bonds, while edges connecting ligand atoms with amino acids denote spatial proximity, suggesting potential non-covalent interactions between them.

Supplementary Figure 4 - OOD Evaluation

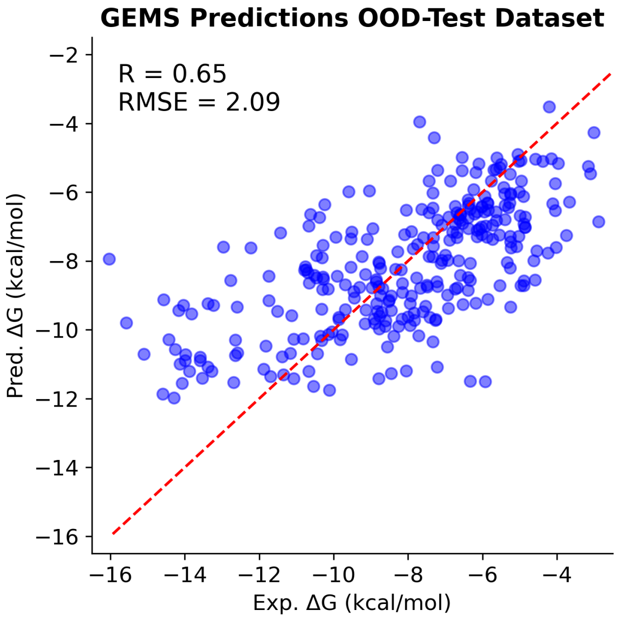

Supplementary Figure 4: Benchmarking of GEMS on out-of-distribution (OOD) test dataset (n=295): Scatterplot of experimental versus predicted binding free energies on the challenging out-of-distribution (OOD) test dataset introduced by Valsson et al. Predictions are obtained with a preliminary version of GEMS trained using 5-fold cross-validation, without further optimization. GEMS achieved a root mean squared error (RMSE) of 2.09 (standard deviation 0.13) and a Pearson correlation of 0.66 (standard deviation 0.06). These results are within the bootstrap confidence intervals reported for AEV-PLIG, despite AEV-PLIG being trained without cross-validation.

Supplementary Note - Training Behaviour Changes with Dataset Filtering

During the training of GEMS, all model variants were subjected to the same five-fold cross-validation procedure. Among all tested variants of GEMS, the variant with the highest and most consistent validation performance across all five folds was selected for testing on the CASF test dataset. Using the cross-validation performance as a selection criteria ensures the choice of the model showing the most robust generalization across different subsets of our training data, making it the most likely to generalize to new data. However, for GEMS models trained on the original PDBbind, the cross-validation performances did not correlate positively with the test set outcomes. Many models with moderate or low cross-validation performance achieved top-tier results on CASF2016 (RMSE of up to 1.15) and CASF2013 (RMSE up to 1.265), which is, to our knowledge, the best performance on CASF2013 reported to date. Despite these excellent benchmark metrics, we disregarded these models and focused on the models with highest cross-validation performance, as these are most likely to generalize successfully to new data.

This discrepancy between cross-validation results and actual test performance is concerning, as 5-fold cross-validation is generally considered a strong indicator of generalization. Notably, these high-performing models achieved their best test results with minimal dropout, whereas increasing dropout improved cross-validation but reduced test performance. This suggests that these models trained on PDBbind rely on overfitting and memorizing training data. Due to the train-test overlap, overfitting to the training data also effectively boosts test dataset performance, resulting in models with exceptional benchmark performance.

In contrast to the models trained on PDBbind, the GEMS models trained on PDBbind CleanSplit showed a closer correlation between 5-fold cross-validation performances and CASF test performance. The models that achieved the highest cross-validation performance consistently showed the best test set results. Additionally, these models typically achieved the highest validation and test performances with higher levels of dropout, highlighting that the prevention of overfitting is crucial for enhancing their test performance. This indicates that GEMS models trained on PDBbind CleanSplit do not rely on memorization, but rather on an understanding of the factors that contribute to high-affinity protein-ligand interactions.

Supplementary Note - Graph Construction and Featurization

To train a robust prediction model on structural protein-ligand data, a sparse, rotation and translation-invariant encoding of the structural data is vital to allow models to learn on this data in a parameter-efficient way. We used graph representations to model the interaction in a protein-ligand complex. The core of these graph representations is an atom-level molecular graph of the ligand molecule, which is extended with an amino acid-level graph representation of the protein pocket. The exclusion of protein residues not involved in ligand binding and the sparse modeling of the protein pocket on amino acid level significantly reduced the size and complexity of the molecular graphs while preserving essential interaction data. In addition, the reduced complexity of the graphs accelarated model training compared to 3D-CNNs and GCN models with more detailed atom-level protein representations.

Nevertheless, the main advantage of representing amino acids as single nodes is the possibility to featurize these nodes with amino acid embeddings derived from protein language models. These models have been trained on vast collections of protein data, making the generated embeddings rich in biological and structural information. We hypothesized that incorporating these amino acid embeddings would significantly increase the predictive power of our models. Indeed, our results demonstrate that models trained on graphs featurized with language model embeddings significantly outperform baseline models that lack these features (see **Figure ??c**). By leveraging these embeddings, we can greatly enhance the feature set for our machine learning tasks, leading to more accurate predictions of protein-ligand binding affinities.

Supplementary Note - Model Architecture

In our research, we employ a graph convolutional network (GCN) architecture designed to efficiently and effectively process graph-structured molecular data. The core model is a graph attention (GAT) network combined with multi-layer perceptrons (MLP) for updating edge features. Initially, node and edge features undergo transformation via MLPs, followed by a sequence of alternating updates for nodes and edges. After each edge feature update, the node features are updated using graph attention network (GATv2Conv) convolution, followed by an update of the global features. This sequence of operations allows our model to capture multi-level information from individual nodes, edges, and the entire graph structure. The global graph features are dynamically updated throughout the process, integrating node representations based on different neighborhood ranges. This combination of detailed local information with broader global information allows the model to integrate both local and global connectivity patterns into the final graph representation. These architectural features make this model setup particularly suitable for molecular graph-level tasks such as protein-ligand binding affinity prediction.
